# Supplementary material for: Artificial Neural Network Prediction of Antiadhesion and Antibiofilm-Forming Effects of Antimicrobial Active Mushroom Extracts on Food-Borne Pathogens
Source: Antibiotics (Basel). 2023 Mar 22;12(3):627. doi: 10.3390/antibiotics12030627 (PMC10045919; doi:10.3390/antibiotics12030627)
Supplement: Supplementary file 1 [file antibiotics-12-00627-s001.zip › Supplementary material.pdf]

Supplementary File

Table S1: The list of mushroom species and their abbreviations

Species	Abbreviation
Meripilus giganteus water extract	MgV
M. giganteus alkali extract	MgA
Agaricus silvaticus water extract	AsV
A. silvaticus alkali extract	AsA
Cratherellus cornucopioides water extract	CcV
C. cornucopioides alkali extract	CcA
Ganoderma applanatum water extract	GaV
G. applanatum alkali extract	GaA
Piptoporus betulinus water extract	PbV
P. betulinus alkali extract	PbA
Laetiporus sulphureus water extract	LsV
L. sulphureus alkali extract	LsA
Schizophyllum commune water extract	ScV
S. commune alkali extract	ScA
Fomitopsis pinicola water extract	FpV
F. pinicola alkali extract	FpA
Boletus edulis water extract	BeV
B. edulis alkali extract	BeA
Clitocybe geotropa water extract	CgV
C. geotropa alkali extract	CgA
Lenzites betulinus water extract	LbV
L. betulinus alkali extract	LbA
Polyporus squamosus water extract	PsV
P. squamosus alkali extract	PsA
Cantharellus cibarius water extract	ChcV
C. cibarius alkali extract	ChcA
Agrocybe aegerita water extract	AaV
A. aegerita alkali extract	AaA
Trametes versicolor water extract	TvV
T. versicolor alkali extract	TvA
Daedalea quercina water extract	DqV
D. quercina alkali extract	DqA
Auricularia mezenterica water extract	AmV
A. mezenterica alkali extract	AmA
Picipes badius water extract	PibV
P. badius alkali extract	PibA
Ganoderma lucidum water extract	GlV
G. lucidum alkali extract	GlA
Fistulina hepatica water extract	FhV
F. hepatica alkali extract	FhA

Table S2. Antiadhesion and antibiofilm-forming activity of water and alkali mushroom extracts against *L. monocytogenes* ATCC 19111 and *S. enteritidis* ATCC 13076

	conc. (mg/mL)	Water extract				Alkali extract			
		L. monocytogenes		*S. enteritidis*		*L. monocytogenes*		*S. enteritidis*	
		%IA	%IB	%IA	%IB	%IA	%IB	%IA	%IB
Mg	0.156	64.37±1.30	93.94±1.90	14.29±2.19	64.21±2.73	43.41±3.41	87.77±3.57	0.00±0.00	70.67±3.10
	0.312	71.26±2.77	93.79±3.19	17.65±1.89	72.84±1.74	47.31±3.16	87.60±0.93	9.24±2.90	77.66±3.08
	0.625	70.06±3.92	89.81±3.30	16.39±1.12	88.41±4.78	28.14±2.77	82.28±3.41	9.80±0.89	0.00±0.00
	1.25	65.97±2.72	94.16±1.15	22.69±2.04	55.56±4.57	41.92±3.48	87.70±1.25	14.29±1.62	0.00±0.00
	2.5	48.70±2.30	69.13±3.37	21.01±1.38	20.99±2.57	43.41±1.48	63.80±2.91	15.97±2.01	0.00±0.00
As	0.156	54.37±1.63	70.83±7.21	21.11±2.24	97.36±4.00	48.74±2.41	27.65±1.33	24.44±2.98	98.34±0.48
	0.312	54.88±1.00	80.76±3.75	22.59±3.07	91.25±1.24	44.63±1.54	57.98±2.46	25.93±3.66	92.98±0.78
	0.625	52.23±1.57	78.29±3.77	25.43±3.95	91.68±1.76	52.51±1.70	69.30±1.49	20.49±1.93	94.89±0.92
	1.25	54.57±2.34	73.18±3.35	18.52±3.92	88.21±1.75	57.53±1.52	50.38±1.18	18.89±1.91	97.08±2.54
	2.5	52.48±2.19	69.78±1.75	9.63±4.64	65.34±0.68	58.22±1.02	44.87±1.58	16.30±3.73	52.57±5.79
Cc	0.156	62.80±1.02	91.63±0.45	4.04±2.09	25.67±0.67	70.34±2.04	68.39±1.00	13.23±2.50	67.59±5.47
	0.312	60.79±1.48	93.29±0.73	4.93±2.56	34.69±1.32	75.99±0.74	67.91±2.63	15.25±3.16	75.86±1.96
	0.625	60.91±2.12	95.31±0.63	1.79±1.69	0.00±0.00	74.99±1.77	60.79±1.16	10.87±1.77	84.95±6.01
	1.25	66.44±1.15	95.40±2.90	0.00±0.00	0.00±0.00	74.17±1.15	65.85±1.19	6.84±2.82	75.21±3.93
	2.5	60.28±0.52	89.98±2.01	0.00±0.00	0.00±0.00	69.59±0.97	0.00±0.00	0.00±0.00	44.71±2.00
Ga	0.156	57.71±1.06	37.90±3.22	29.38±2.27	30.88±2.49	78.30±1.94	61.03±2.84	21.60±3.68	14.78±0.99
	0.312	60.75±2.59	15.13±2.57	27.18±2.37	43.16±2.11	78.94±2.03	55.65±0.91	23.69±3.28	25.07±1.46
	0.625	61.16±3.26	49.61±1.94	24.74±2.36	49.20±1.69	80.82±2.57	84.89±5.06	25.09±4.11	50.70±3.52
	1.25	62.02±1.48	41.42±2.37	28.46±1.13	40.11±2.76	79.02±1.63	79.28±4.00	24.74±3.47	51.56±2.66
	2.5	61.65±4.51	0.00±0.00	11.27±1.79	23.06±2.58	76.97±0.95	0.00±0.00	26.83±3.08	33.18±1.89
Pb	0.156	67.06±4.00	90.78±1.22	16.23±3.40	73.11±2.10	58.08±5.78	84.72±2.95	14.83±1.13	86.16±2.43
	0.312	64.45±1.75	90.22±1.89	16.28±2.53	90.82±2.55	49.31±1.71	84.76±2.53	15.18±2.39	89.08±2.09
	0.625	60.23±4.12	95.11±1.95	19.02±2.10	70.72±5.97	38.63±3.44	94.80±1.82	16.23±2.54	99.21±2.55
	1.25	51.54±5.60	95.04±1.51	19.00±1.33	60.13±4.72	30.02±4.56	88.16±0.92	20.42±1.79	54.39±3.25
	2.5	56.96±2.92	0.00±0.00	0.00±0.00	0.00±0.00	0.00±0.00	0.00±0.00	0.00±0.00	45.02±1.62
Ls	0.156	49.68±1.12	75.82±0.99	0.00±0.00	0.00±0.00	51.61±6.54	73.94±1.97	26.36±1.38	49.91±6.46
	0.312	53.18±3.90	69.67±1.34	0.00±0.00	0.00±0.00	55.46±1.35	68.07±0.63	35.56±2.47	56.96±5.89
	0.625	57.71±3.21	67.16±0.93	0.00±0.00	7.43±2.02	56.17±1.64	69.34±2.84	39.75±2.51	69.43±3.57
	1.25	57.60±7.26	62.96±4.17	0.00±0.00	17.67±1.35	56.96±3.66	54.30±2.81	40.31±2.32	69.80±5.50
	2.5	58.03±3.12	45.67±2.68	0.00±0.00	30.68±1.80	55.03±2.84	84.13±1.76	17.99±1.75	63.32±5.79
Sc	0.156	59.75±2.15	75.54±1.26	3.65±0.49	99.19±3.05	61.72±2.17	87.74±1.25	16.76±1.70	96.19±4.36
	0.312	55.08±1.70	83.36±1.57	1.69±0.68	99.95±2.19	58.79±1.48	88.47±4.16	14.47±1.97	98.39±1.23
	0.625	59.89±0.53	87.08±1.05	0.05±0.18	87.84±1.08	59.75±1.69	94.66±5.63	12.17±1.70	99.92±1.05
	1.25	57.77±2.76	86.39±3.88	0.00±0.00	42.4±1.23	58.47±2.29	99.48±1.73	6.60±2.25	97.72±1.05
	2.5	61.58±3.16	78.263.93	0.00±0.00	33.33±0.91	55.30±0.75	91.49±2.09	0.00±0.00	52.58±2.72
Fp	0.156	49.60±2.14	79.99±1.58	0.00±0.00	73.78±2.67	52.21±3.38	85.73±3.98	9.72±1.12	86.80±2.02
	0.312	50.00±5.82	82.74±2.26	0.00±0.00	82.17±2.61	53.21±4.62	84.00±2.34	7.87±2.34	86.13±1.50
	0.625	41.97±5.00	87.58±1.56	0.00±0.00	96.97±2.21	60.64±3.18	95.16±1.51	2.78±2.06	60.70±1.28
	1.25	42.57±5.17	83.99±1.06	0.00±0.00	98.62±3.72	63.19±2.45	99.67±1.69	0.00±0.00	57.56±2.01
	2.5	30.72±7.53	65.84±5.17	0.00±0.00	62.91±4.13	53.55±0.39	85.43±2.93	0.00±0.00	0.00±0.00
Be	0.156	49.73±0.93	83.99±2.17	9.70±3.32	75.90±4.18	59.81±1.94	88.80±2.08	11.52±3.10	86.42±2.64
	0.312	47.96±1.63	65.59±2.44	1.21±0.98	85.63±3.94	59.74±2.06	97.91±5.00	6.67±1.74	90.18±1.21
	0.625	43.05±1.79	65.65±1.55	0.00±0.00	87.34±1.90	59.35±2.75	74.57±4.36	4.85±3.15	92.41±2.63
	1.25	32.25±4.58	88.81±3.80	0.00±0.00	85.21±2.63	55.20±1.60	73.38±1.36	0.00±0.00	89.61±2.16
	2.5	18.57±3.78	37.97±2.72	0.00±0.00	41.22±0.89	43.09±2.25	26.61±1.94	0.00±0.00	53.26±2.69
Cg	0.156	61.75±1.30	89.23±0.76	9.40±1.45	74.64±1.32	70.35±2.31	87.83±1.58	22.48±1.72	78.42±4.24
	0.312	62.82±2.21	86.69±0.34	0.00±0.00	44.84±1.87	67.36±3.31	90.24±3.06	27.52±3.90	92.11±1.61
	0.625	64.26±1.06	84.71±1.19	0.00±0.00	36.01±0.66	67.60±2.33	90.08±1.22	29.78±3.81	92.54±1.87
	1.25	63.66±1.98	71.97±0.63	0.00±0.00	0.00±0.00	66.47±3.26	95.18±1.25	29.87±3.14	86.29±1.07
	2.5	59.12±1.23	66.66±2.22	0.00±0.00	0.00±0.00	60.31±2.74	89.62±1.78	13.42±3.80	28.00±1.87
	0.156	37.91±1.24	98.49±1.69	24.19±2.38	0.00±0.00	46.06±3.50	95.49±3.60	45.52±2.45	42.90±2.41
	0.312	42.30±2.99	97.08±2.69	36.50±3.86	22.81±0.36	54.84±1.64	87.31±3.97	47.33±0.82	28.45±2.43

Lb	0.625	37.70±3.98	93.50±3.68	45.19±1.29	61.41±2.03	63.41±2.70	78.34±3.96	49.54±3.40	83.94±1.74
	1.25	37.28±2.84	64.91±2.14	49.13±4.55	72.03±5.24	62.51±1.20	49.99±3.42	51.27±4.12	92.57±4.56
	2.5	35.40±2.90	53.15±3.48	38.96±1.19	72.64±0.65	40.21±4.44	0.00±0.00	39.94±2.83	0.00±0.00
Ps	0.156	43.66±2.49	68.55±1.36	11.58±4.53	0.00±0.00	54.45±1.69	90.94±2.83	46.61±4.69	0.00±0.00
	0.312	50.86±3.15	67.98±2.94	18.60±5.25	0.00±0.00	54.45±2.42	90.82±1.43	46.37±1.99	7.15±1.84
	0.625	50.62±0.73	71.04±4.01	24.67±5.45	0.00±0.00	51.10±2.56	96.54±2.67	48.77±2.86	16.92±1.36
	1.25	46.30±2.20	62.42±1.76	24.35±5.23	0.00±0.00	51.00±2.67	99.59±1.70	51.64±2.70	52.83±3.88
	2.5	45.34±3.87	62.72±2.55	21.48±5.65	0.00±0.00	47.02±3.05	99.08±1.83	44.14±4.43	0.00±0.00
Chc	0.156	62.06±1.42	92.66±1.76	18.03±2.71	31.07±3.20	53.23±3.08	86.12±2.82	35.53±1.03	88.28±4.80
	0.312	65.15±1.22	99.96±2.08	21.73±3.32	46.56±2.89	60.17±2.15	91.61±0.67	34.61±3.42	89.37±1.50
	0.625	65.50±1.02	85.30±3.80	30.92±2.91	53.15±0.93	67.63±0.98	87.23±0.73	34.66±3.47	86.82±1.75
	1.25	65.74±2.97	81.98±4.82	20.10±2.66	48.89±1.84	69.24±3.18	69.76±5.01	33.07±1.76	79.89±4.93
	2.5	66.33±1.31	39.43±2.12	0.00±0.00	0.00±0.00	69.77±2.91	51.40±1.46	18.26±0.99	69.62±2.40
Aa	0.156	19.58±4.68	98.21±2.20	15.14±1.33	57.34±2.63	26.98±3.42	97.51±2.55	35.58±1.42	99.25±3.87
	0.312	22.22±3.78	88.94±3.42	9.94±0.77	57.36±6.85	29.37±5.06	99.89±1.21	37.50±1.22	92.36±5.98
	0.625	25.40±2.80	93.48±2.27	0.00±0.00	72.94±5.50	30.95±4.02	99.41±2.94	42.79±1.51	91.96±3.52
	1.25	27.78±3.01	99.71±0.68	0.00±0.00	35.48±2.75	32.54±2.11	98.55±47.89	25.00±0.80	86.78±3.11
	2.5	28.57±3.64	92.35±1.09	0.00±0.00	0.00±0.00	26.98±2.93	47.89±1.66	0.96±0.32	58.69±5.19
Tv	0.156	18.72±2.76	87.98±3.20	9.92±2.49	0.00±0.00	25.55±1.93	88.14±1.66	47.57±3.03	98.80±3.88
	0.312	27.60±3.07	84.80±2.08	19.51±1.57	0.00±0.00	27.60±1.87	73.69±4.55	49.60±1.28	83.51±2.86
	0.625	34.77±1.27	77.37±2.39	43.14±2.53	0.00±0.00	39.89±3.19	67.79±2.79	51.08±2.16	61.48±2.60
	1.25	36.48±2.31	73.00±1.42	53.73±4.09	0.00±0.00	39.21±4.78	49.81±4.72	53.23±2.49	0.00±0.00
	2.5	39.21±1.06	41.64±1.61	58.65±2.07	0.00±0.00	31.69±4.12	32.55±3.18	29.11±3.58	0.00±0.00
Dq	0.156	35.62±1.20	92.13±1.16	9.69±4.20	43.38±1.33	39.98±3.20	59.58±1.97	36.74±3.82	59.53±3.91
	0.312	44.35±1.80	88.91±1.16	27.59±2.06	57.03±1.30	30.71±1.74	79.54±3.99	38.41±3.54	61.55±0.81
	0.625	45.44±1.80	87.05±1.63	30.71±2.62	60.14±3.56	30.89±2.01	86.69±2.38	38.96±2.28	73.12±2.57
	1.25	46.71±2.44	74.68±2.68	40.90±2.42	74.90±4.55	32.89±1.50	90.96±1.38	40.35±4.06	83.27±3.18
	2.5	55.26±2.78	56.64±1.62	3.03±1.43	64.45±0.89	25.25±1.21	11.56±1.08	46.45±3.39	88.25±3.55
Am	0.156	33.33±1.99	59.79±0.77	0.00±0.00	67.80±6.73	48.80±3.80	67.52±2.09	16.55±2.05	85.58±2.45
	0.312	37.60±2.29	63.88±1.25	0.00±0.00	65.95±4.71	49.87±2.40	69.45±2.78	14.63±2.50	81.89±2.91
	0.625	41.87±2.35	64.81±4.55	0.00±0.00	60.70±3.73	58.40±3.52	32.11±1.96	7.43±3.26	79.93±1.90
	1.25	44.80±1.63	82.15±3.83	0.00±0.00	27.63±1.34	61.07±1.85	10.89±3.24	3.60±2.34	67.43±2.02
	2.5	45.60±3.91	30.3±4.82	0.00±0.00	0.00±0.00	53.96±1.85	0.00±0.00	0.00±0.00	0.00±0.00
Pib	0.156	32.73±3.44	56.63±2.31	0.00±0.00	0.00±0.00	40.46±4.96	59.31±4.06	20.69±5.02	52.10±4.93
	0.312	38.74±4.92	58.51±4.30	0.00±0.00	0.00±0.00	44.32±4.81	83.97±1.84	20.89±3.71	77.94±3.07
	0.625	42.18±4.68	76.79±1.60	0.00±0.00	0.00±0.00	45.61±4.32	88.05±3.23	21.84±4.20	48.53±4.93
	1.25	43.32±3.72	86.16±0.95	0.00±0.00	0.00±0.00	46.18±3.02	97.14±1.01	19.16±2.08	61.40±2.47
	2.5	34.73±4.96	42.61±4.31	0.00±0.00	0.00±0.00	47.90±1.74	92.13±3.47	1.53±2.66	32.91±4.90
Gl	0.156	28.04±1.49	43.39±3.56	2.22±0.98	0.00±0.00	25.89±2.10	58.45±4.87	17.26±0.80	53.52±0.91
	0.312	39.73±4.71	64.16±0.79	0.00±0.00	0.00±0.00	26.20±2.67	84.74±1.99	15.56±1.33	87.69±1.23
	0.625	44.24±2.76	55.90±0.9	0.00±0.00	0.00±0.00	32.96±1.87	58.63±2.19	13.50±1.75	79.01±2.02
	1.25	50.49±2.35	46.10±3.40	0.00±0.00	54.92±2.10	40.75±3.87	33.07±1.37	4.27±0.66	84.04±4.75
	2.5	50.80±2.93	33.45±3.34	0.00±0.00	54.30±5.30	46.49±2.42	0.00±0.00	0.00±0.00	86.00±5.97
Fh	0.156	9.72±0.92	67.13±3.15	0.00±0.00	28.64±1.78	15.08±3.84	74.6±3.64	17.16±4.32	74.30±0.48
	0.312	27.92±1.89	76.11±2.42	0.00±0.00	34.57±4.75	16.50±2.92	77.07±2.76	17.16±1.72	85.89±1.53
	0.625	27.56±4.57	67.23±3.17	0.00±0.00	0.00±0.00	17.22±2.64	79.52±1.48	12.04±1.79	79.41±0.68
	1.25	32.91±1.07	28.44±2.96	0.00±0.00	0.00±0.00	25.78±2.89	69.37±1.87	14.37±2.37	64.94±1.59
	2.5	33.87±4.00	0.00±0.00	0.00±0.00	0.00±0.00	26.49±3.05	28.97±2.36	0.00±0.00	0.00±0.00

All experiments were done in four repetitions and the results are presented as the mean values ± standard deviations

Table S3. Elements of matrix W_1 and vector B_1 (presented in the bias row), for ANN model

Input variables	1	2	3	4	5	6	7	8	9
Conc	-0.553	-0.051	-0.746	-0.476	-0.616	0.190	-2.740	-0.684	-0.717
Bact (*L. monocytogenes*)	0.163	1.556	1.060	0.723	1.474	1.662	2.053	2.620	2.203
Bact (*S. enteritidis*)	-1.146	-1.419	-1.203	-0.256	-2.744	-0.517	-0.177	-1.383	-2.630
Funghi (Aa)	0.373	-0.565	2.028	-0.462	0.712	-0.199	-1.013	0.721	-1.090
Funghi (Am)	0.011	0.115	0.114	0.727	-0.938	0.302	-0.149	-0.720	-0.674
Funghi (As)	0.253	-0.709	0.068	0.779	-0.814	2.209	0.187	-0.198	-0.876
Funghi (Be)	0.932	0.429	0.685	-0.226	0.076	0.209	-0.477	-1.086	-0.582
Funghi (Cc)	-0.108	-1.808	-1.015	0.361	-2.253	-0.574	-1.101	-0.522	-0.368
Funghi (Cg)	0.986	-0.796	0.098	-0.003	-0.307	-1.918	-0.751	-0.573	1.358
Funghi (Chc)	0.662	-0.202	-0.473	-0.493	-0.346	-0.088	-0.021	0.189	0.012
Funghi (Dq)	-0.608	-0.677	-0.316	0.711	0.421	1.488	2.359	0.973	-0.985
Funghi (Fh)	-2.230	-0.107	-0.192	-0.288	0.635	-0.860	-0.689	-0.268	-1.042
Funghi (Fp)	0.698	2.361	0.598	0.358	0.027	1.433	-0.018	-1.657	2.085
Funghi (GI)	-2.134	-1.388	-1.071	1.461	0.237	-1.725	1.915	-0.343	-0.674
Funghi (Ga)	0.394	1.893	-1.161	-1.078	-1.005	0.870	1.158	0.138	-0.392
Funghi (Lb)	0.707	-0.125	-0.279	-0.443	1.075	0.645	0.493	1.437	-0.673
Funghi (Ls)	0.470	-0.115	-0.278	0.470	0.034	-0.872	1.870	0.399	0.553
Funghi (Mg)	-0.301	1.824	0.858	-1.234	-0.212	2.763	-2.370	-0.406	1.427
Funghi (PIB)	-0.364	-0.081	-0.015	0.792	0.263	-1.782	1.200	0.060	1.136
Funghi (Pb)	-0.767	-0.490	-0.650	-0.698	0.375	0.788	-2.222	-1.056	-0.667
Funghi (Ps)	0.454	0.414	0.397	-0.365	0.232	-0.986	1.058	1.805	0.210
Funghi (Sc)	0.772	0.587	0.992	0.587	-0.276	-0.305	0.176	-0.921	2.082
Funghi (Tv)	-1.118	-0.547	-0.534	-0.518	0.721	-0.295	0.234	3.168	-1.160
Extract (A)	0.338	0.852	-0.204	-0.045	0.365	2.176	0.602	0.364	-0.137
Extract (V)	-1.341	-0.759	0.053	0.510	-1.634	-0.963	1.295	0.863	-0.270
Bias	-1.017	0.090	-0.167	0.459	-1.232	1.146	1.875	1.236	-0.406

Table S4. Elements of matrix W_2 and vector B_2 (presented in the bias column), for ANN model

Process	1	2	3	4	5	6	7	8	9	Bias
Inhibition of adhesion	0.692	-0.356	-0.353	-0.406	-0.536	0.386	0.145	0.664	0.711	-0.220
inhibition of biofilm-forming	-0.277	-1.608	0.821	-1.178	0.430	1.168	1.435	-1.328	1.222	0.224
